# The Family Health Strategy as a policy of the State: trajectory, institutional capacity and the current primary health care agenda

**DOI:** 10.1590/0102-311XEN271325

**Published:** 2026-06-22

**Authors:** Dirceu Klitzke

**Affiliations:** 1 Ministério da Saúde, Brasília, Brasil.

The article by Giovanella et al. ^1^ provides a comprehensive and timely overview of the 30-year history of the Family Health Strategy (FHS) within Brazilian Unified National Health System (SUS, acronym in Portuguese), emphasizing its role as the cornerstone of primary health care (PHC).

Using the FHS as the central theme, this article highlights how this strategy has been continually shaped by disputes concerning the role of the state, the type of care model and the direction of public policy, repeatedly raising the following central question: Is the FHS intended to support selective PHC focused on providing a narrow scope of services to specific groups, or universal territory-based and comprehensive PHC focused on health needs and addressing the social determinants of health?

The trajectory of the FHS entailed broadening the scope of practices and institutional diversification, which included the incorporation of oral health services and the expansion of multidisciplinarity through the creation of Family Health Expanded Centers (NASF, acronym in Portuguese) and multidisciplinary teams in PHC, alongside specific programs aimed at vulnerable populations, ranging from the homeless to riverine communities.

At the same time, the computerization of PHC progressed with the implementation of e-SUS primary care health record system. With regard to care quality, the Brazilian National Program to Improve Access and Quality in Primary Care (PMAQ-AB, acronym in Portuguese) set standards and created continuous improvement processes, which were later reconfigured as part of the Previne Brasil program and now form part of the Affiliation and Territorial Monitoring and Quality Components of the Current Model.

In terms of infrastructure and facilities, the BHU Upgrade program and the New Growth Acceleration Program for Health supported the construction of new basic health units (BHU) and the purchase of equipment, seeking to improve PHC services physical and technological infrastructure.

In discussion with the authors, my point of departure is the central thesis of this article: the FHS has developed into the cornerstone of PHC in the SUS through a gradual process of expansion driven by federal funding, the municipalization of health care and improvements in work processes, addressing gaps in care.

One of the most sustained achievements over the last 30 years has been the preservation of the integrity of the minimum core of the health team: a doctor, a nurse, a nursing technician and community health workers (CHW). The stability of this “core team” has served as an organizational anchor in the midst of a federative environment characterized by varying municipal government capacities, high levels of staff turnover and changes of government.

The 2006, 2011 and 2017 Brazilian National Primary Health Care Policy (PNAB, acronym in Portuguese) served as milestones, reaffirming commitment to the strategy but also highlighting areas of contention, consolidating family health as the primary model while modifying the scope, coverage and management tools, capturing the tensions between different PHC models.

At the same time, this article provides elements to support the argument that the strategic response to the growing complexity of needs should not be “reforming” the core team, but rather rearguard support and professional development: oral health; matrix support and interprofessional collaboration; evaluation and continuous improvement; and staffing and infrastructure.

In this sense, the historical importance of NASFs/multidisciplinary teams is revealed in the form of an additional political and institutional effect: by providing rearguard technical support and strengthening the clinical capacity of the FHS, these arrangements have helped ease repeated pressure to “reform” minimum staffing levels.

From a federal management perspective, the consolidation of the FHS is the result not only of team structure and normative guidelines but also the existence of a policy ecosystem that provides the physical, technological and institutional conditions for the work carried out by BHU: the PMAQ-AB strengthened evaluation and the development of national quality standards; the BHU Upgrade program and Growth Acceleration Program for Health/New Growth Acceleration Program for Health addressed the historical deficits in infrastructure and computerization. and the More Doctors Program (PMM, acronym in Portuguese) responded to the shortage and uneven distribution of physicians, fostering continuity of care in vulnerable areas.

Between 2016 and 2022, structural policies were replaced by budgetary amendments, cutting funding of the construction and upgrading of BHU facilities and reducing funding for NASF, thus undermining multidisciplinarity. Measures promoting greater flexibility in the role of CHW and alternative arrangements were also introduced, placing strain on the FHS.

We propose to deepen the debate by focusing on territorial institutional capacity: the FHS holds up when it coordinates teams, infrastructure, timely information-sharing that is interoperable across different levels of care and effective network coordination mechanisms. Data from the BHU census reveal weaknesses in information sharing, the recording of patient referral and the monitoring of wait times, limiting the continuity of care.

When these elements remain consistent, the FHS tends to function as a coordinator of care; when they are fragile or discontinuous, the strategy is more vulnerable to changes in the direction of programs and fiscal constraints, even when maintaining high levels of coverage. This perspective helps to inform the discussion of the recent setbacks highlighted in this article. By shifting the focus away from the catchment area population and continuity of care, changes in the financing model and federal incentives have produced effects that go beyond the redistribution of resources. Performance-based payment indicators and mechanisms reorganize work processes, influence care priorities and affect health team-patient/community relationships. Against a backdrop of structural and organizational gaps, these shifts tend to place a strain on the comprehensiveness of care and the capacity of PHC to address the population’s health needs.

Coordination between the FHS and specialized care services remains a key challenge for the SUS and is at the forefront of the Federal Government agenda. Initiatives associated with the Brazilian National Policy on Specialized Health Care (PNAES, acronym in Portuguese) reiterate the need for clear care pathways, effective scheduling and sharing of clinical responsibilities, emphasizing the pivotal role of the FHS in the coordination of care over time ^2^.

Effective coordination requires information infrastructure. In this respect, interoperable electronic health records, integrated scheduling, telehealth and supported decision-making are key components of the coordination capacity of PHC. In the absence of these components, the FHS coordinates care pathways without timely access to information and outcomes, reducing effectiveness and increasing fragmentation.

The national BHU Census plays a key role in providing empirical evidence of inequalities in physical and information infrastructure and multidisciplinary support, informing the debate on continuity of care and network integration and identifying bottlenecks that weaken the capacity of the FHS to effectively coordinate care. At the same time, the census findings reinforce the need to complement this analysis with approaches that incorporate patients’ perspectives through experience-based research and by strengthening public participation mechanisms as an essential dimension of the quality and legitimacy of the SUS.

A qualified health workforce is an essential condition for the effective coordination of care. Employment stability, continuing education and professional development and adequate working conditions foster staff retention, longitudinality and the adoption of collaborative practices and technologies.

While not the focus of this article, the debate on equity cuts across the challenges facing the FHS. Structural racism and other barriers to access interact with territorial factors, gender and social class, producing persistent patterns of health inequalities. Family health teams provide responses based on patient affiliation, but continue to face challenges in institutionalizing practices that address these inequalities.

The trajectory of the FHS urges reflection on governance and institutional arrangements: in a policy guided by territorial accountability, the organization of work processes and services shapes affiliation, longitudinality and coordination of care. In line with Giovanella et al. ^1^, it is argued that strengthening territorial institutional capacity - by integrating PHC, specialized care and information infrastructure - should form the core of the policy.

In this vein, the debate is grounded in the conception of PHC as “*an immense generous gateway to the SUS*” ^3^ (p. 283), supported by an extensive network of local services that are close to patients, universally accessible, provide comprehensive care and have the capacity to address health needs and deliver effective solutions and promote citizenship and health awareness - a vision that continues to guide efforts to address the contemporary challenges in consolidating the FHS within the SUS.

## Data Availability

Not applicable.
