# Supplementary material for: The Family Health Strategy as a policy of the State: trajectory, institutional capacity and the current primary health care agenda
Source: Cad Saude Publica. 2026 Jun 22;42:e00271325. doi: 10.1590/0102-311XEN271325 (PMC13286394; doi:10.1590/0102-311XEN271325)
Supplement: Translation

# Translation: A Estratégia Saúde da Família como política de Estado: trajetória, capacidade institucional e agenda contemporânea da atenção primária à saúde

**DOI:** 10.1590/0102-311XPT271325

**Authors:** Dirceu Klitzke

**Affiliations:** 1 Ministério da Saúde, Brasília, Brasil.

O texto de Giovanella et al. ^1^ oferece uma síntese abrangente e oportuna da trajetória de 30 anos da Estratégia Saúde da Família (ESF) no Sistema Único de Saúde (SUS), reafirmando-a como eixo estruturante da atenção primária à saúde (APS).

Ao tomar a ESF como fio condutor, o artigo evidencia como esse arranjo vem sendo continuamente moldado por disputas em torno do papel do Estado, do modelo de atenção e da direção das políticas públicas, recolocando de forma recorrente uma questão central: estaria a ESF destinada a sustentar uma APS seletiva, focalizada em grupos e de escopo restrito de serviços, ou uma APS abrangente, universal, orientada pelo território, pelas necessidades de saúde e pela determinação social do processo saúde-doença?

Esse percurso combinou ampliação do escopo de práticas e diversificação institucional: incorporação da saúde bucal, expansão da multiprofissionalidade (equipes multiprofissionais - eMulti) via Núcleos Ampliados de Saúde da Família (NASF) e criação de arranjos específicos para populações vulnerabilizadas, da população em situação de rua a comunidades ribeirinhas.

Em paralelo, avançou a informatização da APS com o e-SUS atenção básica/prontuário eletrônico do cidadão. No campo da qualidade, o Programa de Melhoria do Acesso e da Qualidade da Atenção Básica (PMAQ-AB) induziu padrões e processos de melhoria contínua, posteriormente reconfigurados no Previne Brasil e, hoje, nos componentes Vínculo e Acompanhamento Territorial e Qualidade do Modelo Vigente.

No plano da infraestrutura, o Requalifica UBS e o Novo PAC Saúde, ao articular construção de unidades básicas de saúde (UBS) e investimento em equipamentos, buscaram qualificar a base material e tecnológica da APS.

Em diálogo com os autores, tomo como ponto de partida a tese central do ensaio: a ESF consolidou-se como eixo estruturante da APS no SUS por meio de um percurso incremental de expansão, sustentado por indução financeira federal, municipalização, qualificação do processo de trabalho e produzindo respostas para os vazios assistenciais.

Um dos acertos mais consistentes desse ciclo histórico foi preservar, por três décadas, a integridade da equipe nuclear mínima: médico, enfermeiro, técnico de enfermagem e agentes comunitários de saúde (ACS). Essa estabilidade do “núcleo duro” operou como âncora organizativa em um ambiente federativo marcado por heterogeneidades de capacidade estatal municipal, alta rotatividade de profissionais e mudanças de governo.

As Políticas Nacionais de Atenção Básica (PNAB) de 2006, 2011 e 2017 operaram como marcos de reafirmação e arenas de disputa: consolidaram a saúde da família como modelo prioritário, mas também incorporaram ajustes de escopo, cobertura e instrumentos de gestão que refletem tensões entre projetos de APS.

Ao mesmo tempo, o próprio ensaio oferece elementos para sustentar que a resposta estratégica à complexificação das necessidades não deveria recair sobre a “reforma” da equipe mínima, e sim sobre camadas de retaguarda e qualificação: saúde bucal, apoio matricial e multiprofissionalidade, avaliação e melhoria contínua, provimento e infraestrutura.

Nesse sentido, a relevância histórica dos NASF/eMulti também se expressa por um efeito político-institucional adicional: ao oferecer retaguarda técnica e ampliar a potência clínica e sanitária da ESF, esses arranjos contribuíram para mitigar pressões recorrentes de “reforma” da equipe mínima.

Do ponto de vista da gestão pública federal, a consolidação da ESF não decorre apenas do desenho das equipes e de diretrizes normativas, mas de um ecossistema de políticas que assegurou condições materiais, tecnológicas e institucionais ao trabalho nas UBS: o PMAQ-AB fortaleceu a avaliação e a indução de padrões nacionais de qualidade, o Requalifica UBS e o PAC/Novo PAC enfrentaram o déficit histórico de infraestrutura e informatização, e o Programa Mais Médicos mitigou a escassez e a má distribuição de profissionais, ampliando continuidade do cuidado em áreas vulneráveis.

Entre 2016 e 2022, houve substituição de políticas estruturantes por emendas, retraindo investimento contínuo em obras e ampliando passivos nas UBS; desfinanciamento do NASF, enfraquecendo multiprofissionalidade; flexibilizações envolvendo ACS e arranjos alternativos, tensionando atributos da ESF.

Propõe-se aprofundar o debate a partir da capacidade institucional territorial: a ESF se sustenta quando articula equipes, infraestrutura, e capacidade informacional interoperável e oportuna entre níveis de atenção, além de mecanismos efetivos de coordenação em rede. Evidências do Censo das UBS indicam fragilidades no compartilhamento de informações, no registro de encaminhamentos e no acompanhamento de tempos de espera, limitando a continuidade do cuidado em parte expressiva do território.

Onde esses elementos se expressam de forma consistente, a ESF tende a operar como ordenadora do cuidado; onde permanecem frágeis ou descontínuos, a estratégia se torna mais sensível a mudanças de orientação programática e a restrições fiscais, mesmo mantendo elevada cobertura formal. Essa perspectiva contribui para qualificar a discussão sobre os retrocessos recentes apontados no artigo. Mudanças no modelo de financiamento e nos incentivos federais, ao deslocarem o foco da adscrição territorial e da longitudinalidade, produziram efeitos que vão além da redistribuição de recursos. Indicadores e mecanismos de pagamento reorganizam o processo de trabalho, influenciam prioridades assistenciais e afetam a relação das equipes com os usuários e com o território. Em contextos de escassez estrutural e organizacional, esses deslocamentos tendem a tensionar a integralidade do cuidado e a capacidade da APS de responder às necessidades da população.

A coordenação entre APS e atenção especializada permanece desafio estruturante do SUS e ganha centralidade na agenda federal. Iniciativas associadas à Política Nacional de Atenção Especializada em Saúde (PNAES) recolocam a necessidade de fluxos assistenciais claros, regulação efetiva e compartilhamento de responsabilidades clínicas, reafirmando a ESF como nó de articulação do cuidado ao longo do tempo ^2^.

Essa coordenação depende de infraestrutura informacional: prontuários interoperáveis, regulação integrada, telessaúde e apoio à decisão são componentes estruturantes da capacidade coordenadora da APS. Na ausência dessas ferramentas, a ESF coordena trajetórias sem acesso oportuno a informações e resultados, reduzindo resolutividade e ampliando fragmentação.

O Censo das UBS é estratégico por revelar, com base empírica, desigualdades na infraestrutura física e digital e no apoio multiprofissional, qualificando o debate sobre continuidade do cuidado e integração em rede e identificando gargalos que condicionam a função coordenadora da APS. Ao mesmo tempo, seus achados reforçam a necessidade de complementar essa leitura com abordagens que incorporem a perspectiva dos usuários, por meio de pesquisas de experiência e do fortalecimento dos mecanismos de controle social, como dimensão constitutiva da qualidade e da legitimidade do SUS.

A força de trabalho é condição para coordenação do cuidado: estabilidade contratual, formação permanente e condições adequadas de exercício profissional sustentam vínculo, longitudinalidade e adoção de práticas colaborativas e tecnologias.

Ainda que não constitua eixo central do ensaio debatido, a discussão sobre equidade atravessa de forma transversal os desafios da ESF. O racismo estrutural e outras barreiras de acesso interagem com o território, gênero e classe social, produzindo padrões persistentes de desigualdade em saúde. As equipes de saúde da família (eSF) oferecem respostas baseadas em vínculo, mas seguem desafiadas a institucionalizar práticas que enfrentem essas iniquidades.

A trajetória da ESF impõe reflexão sobre governança e arranjos institucionais: numa política orientada pela responsabilização territorial, a organização do trabalho e dos serviços conforma vínculo, longitudinalidade e coordenação do cuidado. Em diálogo com Giovanella et al. ^1^, afirma-se que fortalecer a capacidade institucional territorial, articulando integração APS-atenção especializada e infraestrutura digital, deve estruturar o núcleo da política.

Nessa direção, o debate se articula com uma concepção de APS que se projeta como “*imensa e generosa porta de entrada para o SUS*” ^3^ (p. 283), sustentada em uma rede capilarizada de serviços territoriais, próxima dos usuários, de acesso universal, com capacidade resolutiva, produtora de cuidado integral, promotora de cidadania e de consciência sanitária, horizonte que segue orientando os desafios contemporâneos da consolidação da ESF no SUS.

## Data Availability

Não se aplica.
